# Diagnostic Challenges of Aortic Dissection at 5200m- A Case Report Presenting as Neck and Back Emphysema

**DOI:** 10.2174/0115734056409777251017072912

**Published:** 2025-11-05

**Authors:** XiaoBo Han, Qiong Zhu, XiaoQin Chen, JiaLin Wu, JingDu Tian, Yong Wang, Qian Yang, JianBo Huang, Xi Yang, ZhiXin Gan

**Affiliations:** 1 Department of Emergency, General Hospital of Xinjiang Military Command, Urumqi, China; 2 Department of Ultrasound, The 953rd Hospital of the Chinese People's Liberation Army, Shigatse, China; 3 Department of Ultrasound, Xinqiao Hospital, Army Medical University, ChongQing, China; 4 Department of Pneumology, General Hospital of Xinjiang Military Command, Urumqi, China; 5 Department of Gastroenterology, General Hospital of Xinjiang Military Command, Urumqi, China; 6 Department of Ultrasound, The 950rd Hospital of the Chinese People's Liberation Army, YeCheng, China; 7 Department of Cardiovascular Medicine, General Hospital of Xinjiang Military Command, Urumqi, China; 8 Department of Scientific Research Section, General Hospital of Xinjiang Military Command, Urumqi, China

**Keywords:** Acute aortic dissection, High altitude, Subcutaneous emphysema, High mortality rate, Atypical chest pain

## Abstract

**Background:**

Acute Aortic Dissection (AD) is of great concern due to its high mortality rate. The probability of young patients without underlying diseases developing acute aortic dissection is relatively low. In extreme regions such as high-altitude areas, for patients presenting with atypical chest pain, it is necessary to not only consider life-threatening diseases such as aortic dissection and acute coronary syndrome, but also to rule out the interference of emphysema in the diagnosis. This case provides experience in the diagnosis, evacuation, and treatment of aortic dissection patients in high-altitude areas.

**Case Presentation:**

We present the case of a young man who experienced sudden neck pain at an altitude of 5200 m during defecation. The pain persisted and radiated to the back, but there were no typical symptoms of aortic dissection. However, on physical examination, the patient was found to have unequal blood pressure in both arms. After completing a CT scan, the diagnosis was confirmed as aortic dissection with subcutaneous emphysema. The patient was transferred to a hospital at a lower altitude to undergo an “aortic arch replacement under cardiopulmonary bypass.” After follow-up, the patient had a good prognosis and was able to independently perform general daily activities.

**Conclusion:**

The purpose of this case report is to raise awareness of the diagnostic interference caused by subcutaneous emphysema and to emphasize accurate diagnosis and timely intervention when encountering patients with atypical chest pain in high-altitude environments, which is expected to gain a therapeutic time window for the patient.

## 1 INTRODUCTION

Acute aortic syndrome includes acute aortic dissection, vascular wall hematoma, aortic penetrating ulcer, and thoracicaortic rupture, among which aortic dissection is the most lethal. In patients with undiagnosed Acute Aortic Dissection (AAD), the mortality rate approaches 75% in the second week after onset, and the mortality rate rises to 90% three months later without appropriate treatment [1]. Some studies have shown that male gender, advanced age, hypertension, history of cardiac/valvular surgery, and Marfan syndrome are high-risk factors for the occurrence of aortic dissection. In young people under the age of 40, the incidence of aortic dissection is about 10%, and most of these patients have a history of cardiac valvular surgery, Marfan syndrome, Ehlers-Danlos syndrome, or similar conditions. The risk of aortic dissection in young patients without underlying diseases is very low, and the symptoms of chest pain in these patients are also atypical. It is necessary to correctly identify and diagnose, and treat such patients at an early stage [2, 3].

In high-altitude environments, the unique low-pressure and low-oxygen conditions, combined with limited diagnostic facilities, pose significant challenges to accurate diagnosis. Healthcare workers in primary care settings may encounter patients with atypical chest pain and, due to the lack of CT scans, readily diagnose spontaneous subcutaneous emphysema or pneumomediastinum, opting for conservative treatment measures. This approach can delay the diagnosis and treatment of critical cardiovascular emergencies such as aortic dissection and pulmonary embolism. The impact is further exacerbated by the presence of high-altitude pulmonary edema. In high-altitude regions, where emphysema is more prevalent and often concealed, it is crucial to closely monitor its potential to mask life-threatening conditions.

To our knowledge, there are currently few reports of aortic dissection occurring at high altitudes, and none with subcutaneous emphysema. We are the first to propose aortic dissection with subcutaneous emphysema at high altitudes. This case report is expected to provide important insights into the diagnosis and treatment of such diseases.

## 2 CASE PRESENTATION

**Fig. (1):**
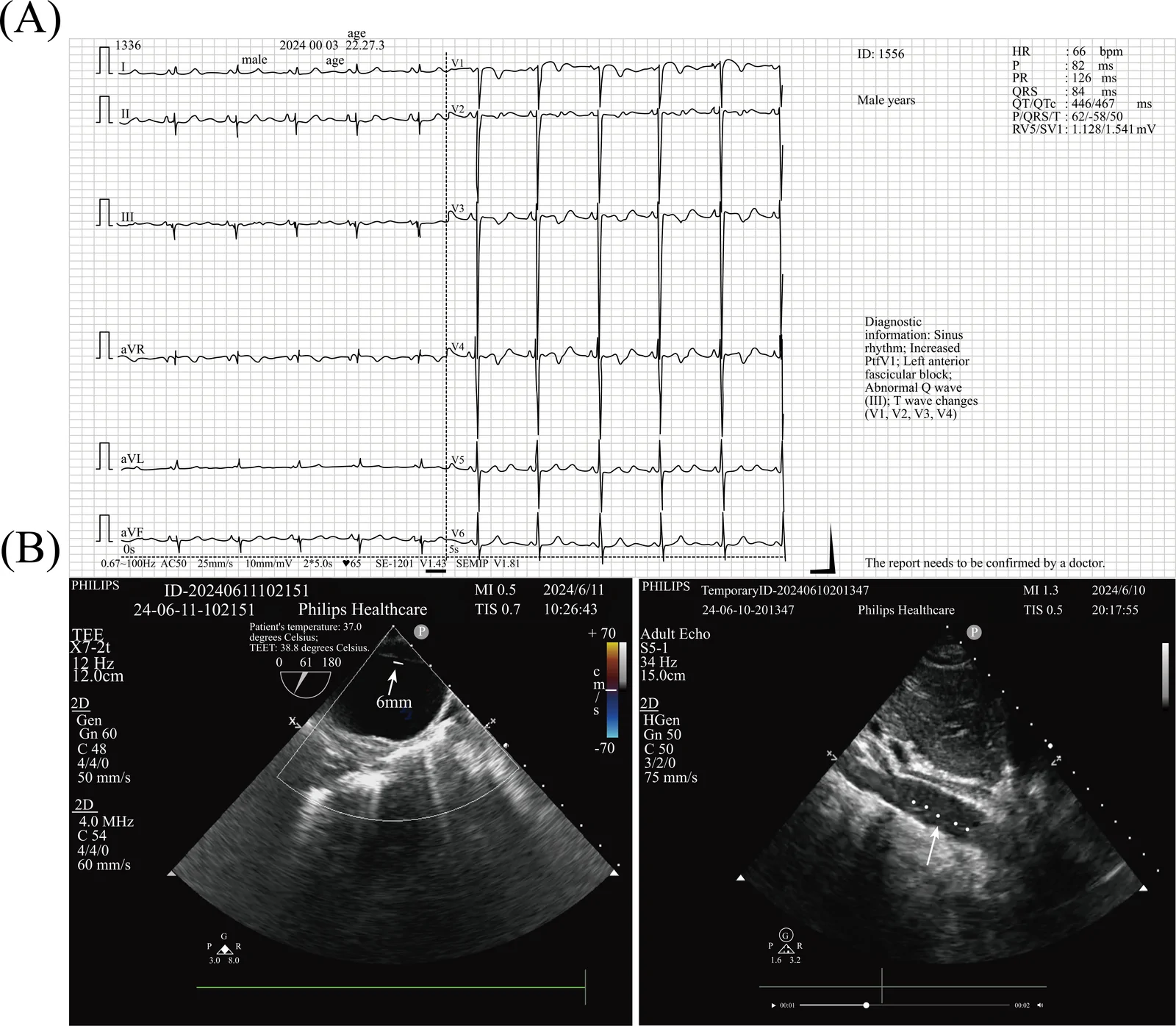
Electrocardiography revealed “sinus rhythm, left axis deviation, left anterior fascicular block and T-wave changes in the anterior leads.” (**A**); The ultrasound showed that the white arrow indicates a 6mm intimal tear (**B**); The dotted line is the interlayer line between true and false lumen (**C**).

**Fig. (2):**
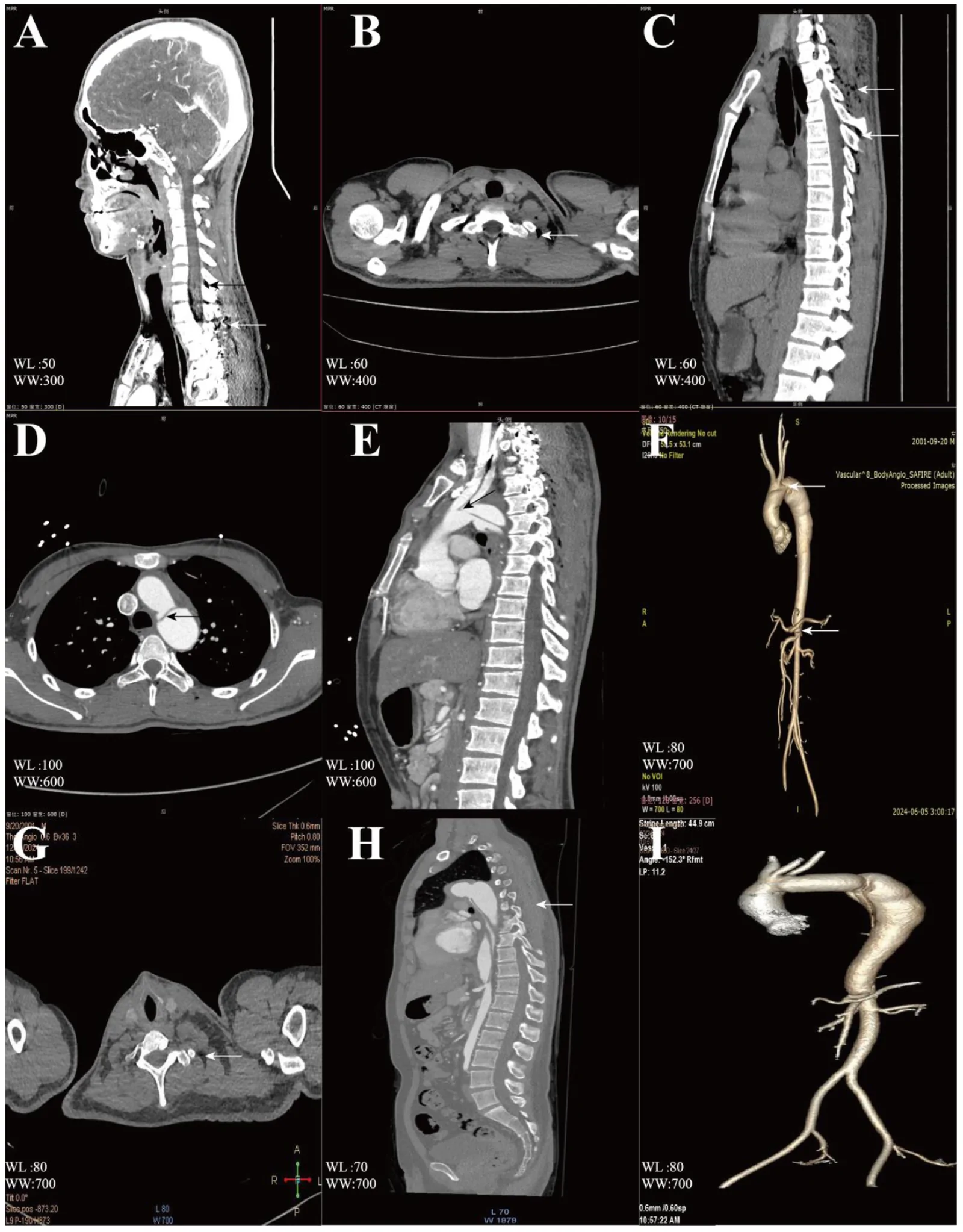
In CT scans, WL means window level and WW means window width. The presence of emphysema in the neck(between the two spinous processes (**A**), beside of transverse process (**B**), and intermuscular and subcutaneous air in the back (**C**); CT angiography of the thoracic and abdominal aorta: The descending aorta showed a double lumen image with a smaller true lumen and larger false lumen (**D**). The intimal rupture is located in the aortic arch, between the left common carotid artery and the left subclavian artery, and the width of the rupture is about 7mm (**E**). The dissection extends down to the upper part of the abdominal aorta, above the opening of the superior mesenteric artery; The left common carotid artery is of common origin with the brachiocephalic artery (**F**).The emphysema disappeared in the neck (**G**) and back (**H**). The 3D reconstruction showed that the artificial blood vessel healed well (**I**).

A 22-year-old man experienced sudden neck pain at an altitude of 5200 m during defecation. The pain persisted and radiated to the back, causing difficulty in standing upright. The patient reported no personal or family history of hypertension. His initial blood pressure was 200/110 mmHg, prompting the administration of 10 mg of nifedipine, but this treatment proved ineffective. After six hours, the patient was transferred to the emergency department at an altitude of 3600 m, where paramedics recorded blood pressures of 202/103 mmHg (right arm) and 190/95 mmHg (left arm). Electrocardiography revealed “sinus rhythm, left axis deviation, left anterior fascicular block and T-wave changes in the anterior leads” (Fig. **1A**). Non-enhanced CT revealed emphysema in the neck and back, with a widening of the aortic arch, descending aorta, and abdominal aorta. Laboratory tests showed a white blood cell count of 12.9×10^9^/L, and D-dimer levels of 1.6 μg/mL. Cardiac troponin I, triglycerides, and cholesterol levels were normal. Physical examination revealed tenderness of the neck and back, prompting suspicion of aortic dissection. The ultrasound showed that the white arrow indicates a 6mm rupture (Fig. **1B**), the dotted line is the score line between true and false lumen (Fig. **1C**), and the flapping shadow of the vascular intima (S1). Non-enhanced CT revealed emphysema in the neck and intermuscular and subcutaneous air in the back (Fig. **2A**-**C**). An enhanced CT scan confirmed Thoracoabdominal aortic dissection. The initial tear located in the aortic arch between the left common carotid and subclavian arteries extended into the upper abdominal aorta (Fig. **2D**-**E**). Notably, the left common carotid and brachiocephalic arteries shared a common trunk (Fig. **2F**). Prompt endovascular repair was performed by the Cardiac Surgery Department of the regional medical center.

After follow-up, it was learned that the patient was transferred to the general ward on the 9th day after surgery. He was able to engage in appropriate out-of-bed activities half a month after the operation, had sutures removed at 2 months, and eventually performed general daily activities. The most recent follow-up was in December 2024. Following CT and other examinations, it was found that the emphysema had been largely absorbed (Fig. **2G**-**H**), and 3D reconstruction showed that the artificial blood vessel had healed well (Fig. **2I**). Throughout the patient’s entire disease course, there was a mild pulmonary infection, which completely resolved after appropriate antibiotic treatment.

## 3 DISCUSSION

Approximately 400 million people live in high-altitude areas above 1500 meters, and more than 40 million people reside at altitudes above 2500 meters. The low atmospheric pressure, hypoxia, low temperature, and intense ultraviolet radiation in plateau regions have a significant impact on human activities. All these factors contribute to the progression of plateau diseases, with hypobaric hypoxia being the most important cause. Oxygen is essential for cellular metabolism, and the hypoxic environment of high altitudes can significantly affect the cardiovascular system. When the human body enters high-altitude regions, the increased activity of the sympathetic nervous system and the secretion of catecholamines in the circulation lead to an increased heart rate at rest. Although prolonged exposure to high-altitude areas increases the activity of the parasympathetic nervous system, leading to a decrease in maximum heart rate, the heart rate remains higher compared to that in plain areas. The increased heart rate results in a reduced left ventricular end-diastolic volume, impaired ventricular filling, and consequently, diastolic dysfunction. The increased myocardial oxygen demand can trigger other diseases [4, 5].

Hypertension is a major risk factor for adverse cardiovascular events and is closely related to diseases such as aortic dissection. Both healthy individuals and hypertensive patients experience the dual effects of local vasodilation from hypoxia and overall sympathetic nerve excitation-induced vasoconstriction when exposed to high-altitude areas. The impact of these two factors on local perfusion and systemic arterial pressure varies greatly among individuals. Some individuals experience an increase in blood pressure when entering plateau areas, while others tend to have stable or even decreased blood pressure. The mechanisms underlying these differences may be related to the regulation of gene expression [6, 7]. Long-term exposure to a hypoxic environment can lead to an increase in red blood cell count and damage to the vascular endothelium. These changes can result in slower blood flow, increased blood viscosity, and elevated pulmonary artery pressure, which may ultimately lead to problems such as thrombosis and heart failure. Additionally, at high altitudes, the increased oxygen demand of the myocardium, combined with an active Renin-Angiotensin-Aldosterone System (RAAS), as well as multiple factors including hypoxia, respiratory alkalosis, and electrolyte imbalances, can lead to life-threatening conditions such as arrhythmias, coronary heart disease, and sudden cardiac death. However, there are a few reports of aortic dissection occurring at high altitudes [8].

In this report, we describe a novel case of aortic arch dissection at high altitudes. The patient is a young male with no family history of hypertension or predisposing factors for aortic dissection, such as Marfan syndrome. Prior to the onset of symptoms, the patient had not engaged in strenuous activity and only experienced mild to moderate neck and back pain after straining during bowel movements, with a pain score of 3. Upon diagnosis, an initial ECG test revealed changes in the anterior wall T wave. However, normal levels of myocardial calcium protein I and the myocardial enzyme spectrum ruled out the possibility of acute coronary syndrome. During the physical examination, we found that the patient had elevated blood pressure on one side. Further measurement of blood pressure on both sides revealed an inequality, which led us to consider the diagnosis of aortic dissection. After completing CT and other examinations, the diagnosis of thoracoabdominal aortic dissection and subcutaneous emphysema was confirmed. We promptly transferred the patient to a lower-altitude hospital under strict control of heart rate and blood pressure, buying valuable time for surgery. Follow-up revealed that the patient is now able to perform daily activities, and the subcutaneous emphysema has been completely absorbed.

In high-altitude areas, medical resources are relatively scarce. Some medical institutions may lack equipment such as CT scanners, and when encountering similar patients, they often only perform chest X-ray examinations and diagnose subcutaneous emphysema. Subcutaneous emphysema can explain the mild neck and back pain experienced by the patient, and with normal laboratory indicators, life-threatening diseases may be overlooked. In our literature review, we found only two reports on aortic dissection in high-altitude areas, focusing on surgical anesthesia considerations and cases in alpine skiing populations. There are currently no reports on aortic dissection with subcutaneous emphysema in extreme high-altitude environments. Additionally, our literature search revealed that aortic dissection in plain areas can be accompanied by CT manifestations such as pleural effusion, interlobular septal thickening, atelectasis due to compression, air trapping, and emphysema, but no reports of concurrent subcutaneous emphysema were found. This further highlights the novelty of our case report [9-11].

Subcutaneous emphysema is commonly seen in the context of iatrogenic procedures, pulmonary diseases, and rupture of the trachea/bronchi or esophagus. In addition to these, infections caused by gas-forming organisms, decompression sickness, or perforation of the gastrointestinal tract can also lead to the development of emphysema. The pathogenesis of this condition can be explained by the Macklin effect. First, an increase in intrathoracic pressure leads to elevated alveolar pressure and alveolar expansion, while the pressure in the vascular lumen remains normal. This ultimately creates a pressure difference between the alveoli and the vessels, causing alveolar rupture and resulting in emphysema. This phenomenon is common in situations such as coughing, vomiting, sneezing, and defecation. Second, when the diameter of the pulmonary vessels decreases while the alveolar pressure remains normal, a pressure difference is generated, causing gas to leak into the perivascular and peribronchial sheaths. This situation is common in cases of airway stenosis or obstruction due to various causes [12, 13]. Further literature search revealed that in plateau environments, the onset of emphysema is younger and more often caused by strenuous activities, which may be related to the unique conditions of these areas. In order to adapt to the plateau environment, the body will produce a large number of red blood cells, which increases the pressure in the circulatory system and leads to cardiopulmonary dysfunction. Meanwhile, the hypoxic environment will directly damage the vascular endothelium, causing pulmonary artery remodeling and pulmonary hypertension, which undoubtedly increases the pressure gradient. Finally, the plateau environment will deepen and accelerate breathing, while over-ventilation will increase alveolar pressure, potentially leading to alveolar rupture and causing emphysema [14-17]. It should be noted that in such cases, the gas first disseminates to the mediastinum, then spreads along the fascial planes to the neck or back, ultimately forming subcutaneous emphysema in the neck or back. Moreover, in such cases, there may be more air in the neck region. However, in this case, the patient had no obvious gas in the mediastinum, and the gas in the back appeared to be more, mostly concentrated in the paravertebral space. Since no cases of aortic dissection combined with subcutaneous emphysema in high-altitude areas have been reported in the literature, and due to the lack of medical resources in these areas, we were unable to dynamically monitor the patient's imaging data. Therefore, based on the existing data, we can only speculate that the occurrence of this phenomenon may be related to the low-pressure environment of the plateau or idiopathic paravertebral emphysema.

We present an atypical case of aortic dissection in an extreme environment, which provides important insights for clinical practice. In the special environment of high-altitude areas, when encountering patients with atypical chest pain, it is essential to conduct relevant examinations as early as possible to rule out life-threatening diseases such as aortic dissection, acute coronary syndrome, and pulmonary embolism. It is also crucial to establish a diagnosis promptly and provide appropriate treatment. For example, in this case, if anticoagulants and antiplatelet drugs had been blindly administered after observing T-wave changes on the ECG as treatment for acute coronary syndrome, it could have led to fatal bleeding. Moreover, in the special environment of high-altitude areas, the occurrence of subcutaneous emphysema may affect the diagnosis of cardiovascular diseases and requires special attention. In summary, this report offers valuable insights for the diagnosis of similar cases.

## 4 LIMITATIONS OF THE STUDY

The limitations of this study lie in the insufficient dynamic imaging data, which preclude detecting the dynamic evolution of emphysema and dissection.

## CONCLUSION

The unique environment of high-altitude areas increases the complexity of diagnosis. When encountering patients with atypical chest pain, it is essential to be vigilant, rule out the interference of subcutaneous emphysema, and make an accurate diagnosis and treatment.

## Data Availability

All data generated or analyzed during this study are included in this published article.

## LIST OF ABBREVIATIONS

AD: Acute Aortic Dissection
AAD: Acute Aortic Dissection
SE: Subcutaneous Emphysema
RAAS: Renin-Angiotensin-Aldosterone System

## STANDARDS OF REPORTING

CARE guidelines were followed.

## References

[1] De León Ayala I.A., Chen Y.F. (2012). Acute aortic dissection: An update.. Kaohsiung J. Med. Sci..

[2] Chukwu M., Ehsan P., Aburumman R.N., Muthanna S.I., Menon S.R., Vithani V., Sutariya B., Montenegro D.M., Mohammed L. (2023). Acute Stanford Type A Aortic Dissection: A review of risk factors and outcomes.. Cureus.

[3] Pape L.A., Awais M., Woznicki E.M., Suzuki T., Trimarchi S., Evangelista A., Myrmel T., Larsen M., Harris K.M., Greason K., Di Eusanio M., Bossone E., Montgomery D.G., Eagle K.A., Nienaber C.A., Isselbacher E.M., O’Gara P. (2015). Presentation, diagnosis, and outcomes of acute aortic dissection.. J. Am. Coll. Cardiol..

[4] Mallet R.T., Burtscher J., Richalet J.P., Millet G.P., Burtscher M. (2021). Impact of high altitude on cardiovascular health: Current perspectives.. Vasc. Health Risk Manag..

[5] Richalet J.P., Hermand E., Lhuissier F.J. (2024). Cardiovascular physiology and pathophysiology at high altitude.. Nat. Rev. Cardiol..

[6] Keyes L.E., Sallade T.D., Duke C., Starling J., Sheets A., Pant S., Young D.S., Twillman D., Regmi N., Phelan B., Paudel P., McElwee M., Mather L., Cole D., McConnell T., Basnyat B. (2017). Blood pressure and altitude: An observational cohort study of hypertensive and nonhypertensive Himalayan Trekkers in Nepal.. High Alt. Med. Biol..

[7] Tremblay J.C., Ainslie P.N., Turner R., Gatterer H., Schlittler M., Woyke S., Regli I.B., Strapazzon G., Rauch S., Siebenmann C. (2020). Endothelial function and shear stress in hypobaric hypoxia: Time course and impact of plasma volume expansion in men.. Am. J. Physiol. Heart Circ. Physiol..

[8] Riley C.J., Gavin M. (2017). Physiological changes to the cardiovascular system at high altitude and its effects on cardiovascular disease.. High Alt. Med. Biol..

[9] Zheng H., Wei X.C., Yu T., Lei Q. (2020). Anesthesia of a high-altitude area inhabitant who underwent aortic dissection emergency surgery in a low-altitude area.. J. Int. Med. Res..

[10] Schachner T., Fischler N., Dumfarth J., Bonaros N., Krapf C., Schobersberger W., Grimm M. (2013). Aortic dissection type A in alpine skiers.. BioMed Res. Int..

[11] Semiz-Oysu A., Okur A., Sahin S. (2012). Pulmonary multislice computed tomography findings in acute aortic dissection.. J. Thorac. Dis..

[12] Macklin C.C. (1937). Pneumothorax with Massive Collapse from Experimental Local Over-inflation of the Lung Substance.. Can. Med. Assoc. J..

[13] Murayama S., Gibo S. (2014). Spontaneous pneumomediastinum and Macklin effect: Overview and appearance on computed tomography.. World J. Radiol..

[14] Rieger M.G., Tallon C.M., Perkins D.R., Smith K.J., Stembridge M., Piombo S., Radom-Aizik S., Cooper D.M., Ainslie P.N., McManus A.M. (2022). Cardiopulmonary and cerebrovascular acclimatization in children and adults at 3800 m.. J. Physiol..

[15] Talbot N.P., Balanos G.M., Dorrington K.L., Robbins P.A. (2005). Two temporal components within the human pulmonary vascular response to ~2 h of isocapnic hypoxia.. J. Appl. Physiol..

[16] Moudgil R., Michelakis E.D., Archer S.L. (2005). Hypoxic pulmonary vasoconstriction.. J. Appl. Physiol..

[17] Stenmark K.R., Fagan K.A., Frid M.G. (2006). Hypoxia-induced pulmonary vascular remodeling: Cellular and molecular mechanisms.. Circ. Res..

